# circRNAs: Insight Into Their Role in Tumor-Associated Macrophages

**DOI:** 10.3389/fonc.2021.780744

**Published:** 2021-12-01

**Authors:** Saili Duan, Shan Wang, Tao Huang, Junpu Wang, Xiaoqing Yuan

**Affiliations:** ^1^ Department of Pathology, Xiangya Hospital, Central South University, Changsha, China; ^2^ Xiangya School of Medicine, Central South University, Changsha, China; ^3^ Department of Pathology, School of Basic Medicine, Central South University, Changsha City, China; ^4^ National Clinical Research Center for Geriatric Disorders, Xiangya Hospital, Central South University, Changsha, China; ^5^ Guangdong Provincial Key Laboratory of Malignant Tumor Epigenetics and Gene Regulation, Sun Yat-Sen Memorial Hospital, Sun Yat-Sen University, Guangzhou, China; ^6^ Breast Tumor Center, Sun Yat-Sen Memorial Hospital, Sun Yat-Sen University, Guangzhou, China

**Keywords:** circRNA, TAMs, M2 macrophage, polarization, cancer

## Abstract

Currently, it is well known that the tumor microenvironment not only provides energy support for tumor growth but also regulates tumor signaling pathways and promotes the proliferation, invasion, metastasis, and drug resistance of tumor cells. The tumor microenvironment, especially the function and mechanism of tumor-associated macrophages (TAMs), has attracted great attention. TAMs are the most common immune cells in the tumor microenvironment and play a vital role in the occurrence and development of tumors. circular RNA (circRNA) is a unique, widespread, and stable form of non-coding RNA (ncRNA), but little is known about the role of circRNAs in TAMs or how TAMs affect circRNAs. In this review, we summarize the specific manifestations of circRNAs that affect the tumor-associated macrophages and play a significant role in tumor progression. This review helps improve our understanding of the association between circRNAs and TAMs, thereby promoting the development and progress of potential clinical targeted therapies.

## Introduction

With the continuous development of high-throughput sequencing, a class of covalently closed RNA molecules with extensiveness, diversity, stability, and evolutionary conservation has come into view, known as circular RNA (circRNA) (1, 2). circRNA has a unique covalently closed loop structure and a specific tertiary structure and exhibits tissue- and developmental stage-specific expression, which plays an essential role in multiple cellular processes (3). Some circRNAs have been reported to be involved in tumor genesis, progression, and metastasis (4, 5), and circRNAs have been identified as important markers of various tumors (6–9). As a critical determinant of all stages of cancer development and progression, the tumor microenvironment (TME) is a complex ecosystem involving the coevolution of cancer cells and the surrounding matrix (10). A variety of cellular components in TME include immune cells [T cells, tumor-associated macrophages (TAMs), dendritic cells, mast cells, *etc.*], cancer-associated endothelial cells (CAE), cancer-associated fibroblasts (CAFs), and cancer stem cells (11, 12). Non-cellular counterparts include growth factors, cytokines, and extracellular matrix (ECM) (13). Other studies have shown that circRNAs play a variety of roles in the TME, promote or inhibit the immune system and angiogenesis, improve the permeability of endothelial cells, promote tumor metastasis, lead to ECM remodeling, and jointly support tumor progression (14, 15)—for example, CAFs can release circEIF3k under hypoxia, upregulate miR-214, and downregulate the programmed death ligand-1 (PD-L1) expression in colorectal cancer, thus inhibiting the progression of colorectal cancer (16). CAF-derived cytokines promote the progression and metastasis of hepatocellular carcinoma (HCC) by activating the circRNA–miRNA–mRNA axis in tumor cells (17). The high expression of cerebellar degeneration-related 1 antisense (circ-CDR1as) can enhance the penetration level of CAEs to promote tumor growth and metastasis (18). circRNAs may become the entry point of the entire ncRNA network, providing broad prospects for the clinical treatment of tumors (19).

Currently, circRNAs participate in the progression of tumorigenesis by acting on the TME and affecting the polarization of TAMs. However, the relationship and interactions of circRNAs and TAMs have not been systematically summarized. In this review, we will outline the specific manifestations of circRNAs affecting the tumor microenvironment as well as the latest findings suggesting that they participate in the metabolic reprogramming of tumor-associated macrophages and play an important role in tumor progression. Our review will improve the understanding of the relationship between circRNA and TAMs to promote the development and progress of potential clinical targeted therapies.

### circRNAs

circRNAs are single-stranded RNAs with covalently closed circular structures with tissue/developmental stage-specific expression patterns (20–23), which are highly regulated by cis-acting elements and trans-acting factors (24–27). The covalently closed loops formed by circRNAs are produced by the back-splicing of the exon and/or intron sequences of the primary transcript and endow them with the inherent ability to resist the decay of extranuclear RNA (28). Back-splicing is catalyzed by the standard spliceosome mechanism, but protein factors and CIS-complementary sequences, especially Alu repeats, can regulate this process (29). Alu complementary-dependent base-pairing supports the connection of downstream splicing donor pairs with non-splicing upstream splicing receptors, and the contributed RNA is covalently closed (30). circRNAs exist in a wide range of species, ranging from viruses to mammals, and can function as transcriptional regulators, microRNA (miR) sponges, and protein templates (6, 31–33). Based on the diversity of source sequences, circRNAs can be divided into three categories: exonic circRNAs (EcRNA), exon–intron circRNAs (EIciRNAs), and circular intronic RNAs (ciRNAs) (8, 29, 34–36). However, a fourth tricRNA may be isolated, which corresponds to intronic circular tRNA (37). Most circRNAs are derived from pre-mRNA, while a small portion of intron-derived circRNAs are derived from pre-tRNA (36, 38).

Many studies have shown that circRNA has several characteristics, namely (22):

(1) Abundance and diversity: thousands of different circRNAs have been identified in eukaryotes through RNA-seq technology, and the complexity of the circRNA production mechanism leads to its diversity (29, 39). The enrichment of circRNA can also be found in saliva and blood (22).

(2) Stability: a unique ring structure makes circRNA resistant to ribonuclease, without 5′–3′ polarity and a polyadenylated tail, which results in higher stability than linear RNA (22).

(3) Conservation: circRNAs are highly conserved in different species (40).

(4) Specificity: circRNAs are usually specifically expressed in a tissue or developmental stage-specific manner (8, 41). The characteristics of circRNAs give them the following different functions:

(a) They have miRNA sponges, such as circRNA sex-determining region Y (cir-SRY) (42). (b) They interact with proteins and their expression, such as retinol-binding proteins and mannose-binding lectins (43).

(c) They have translation templates, such as circRNA zinc-finger protein 609 (circ-ZNF609) (44).

(d) They have transcription regulators, such as circRNA poly(A) binding protein-interacting protein 2 (circ-PAIP2) (45).

circRNAs are generated in the nucleus, but most of them are found in the cytoplasm—for example, circRNAs formed by exons are generally located in the cytoplasm (22), which suggests specific rules for circRNA transport or localization. Although most circRNAs are located primarily in the cytoplasm, ciRNAs and EIciRNAs are limited to the nucleus (23, 35, 46), which means that their role is in nuclear events, such as transcriptional regulation. ciRNAs regulate the transcription of their parental genes by promoting the elongation of polymerase II. The binding of circRNAs to proteins may depend not only on nucleotide sequences but also on the different secondary or tertiary structures of circRNAs (47). Abnormally regulated circRNAs play a suppressive or carcinogenic role in the initiation and progression of cancer, affecting a number of cellular functions, such as the maintenance of proliferation signals, promotion of cell migration and invasion, resistance to apoptosis, and induction of angiogenesis (48, 49). Meanwhile, circRNAs play an important regulatory role in diseases by interacting with disease-related miRNAs (50). Studies have shown that circRNAs are helpful for the treatment of osteoporosis, which is related to the differentiation of osteoclasts (51).

**Figure 1:**
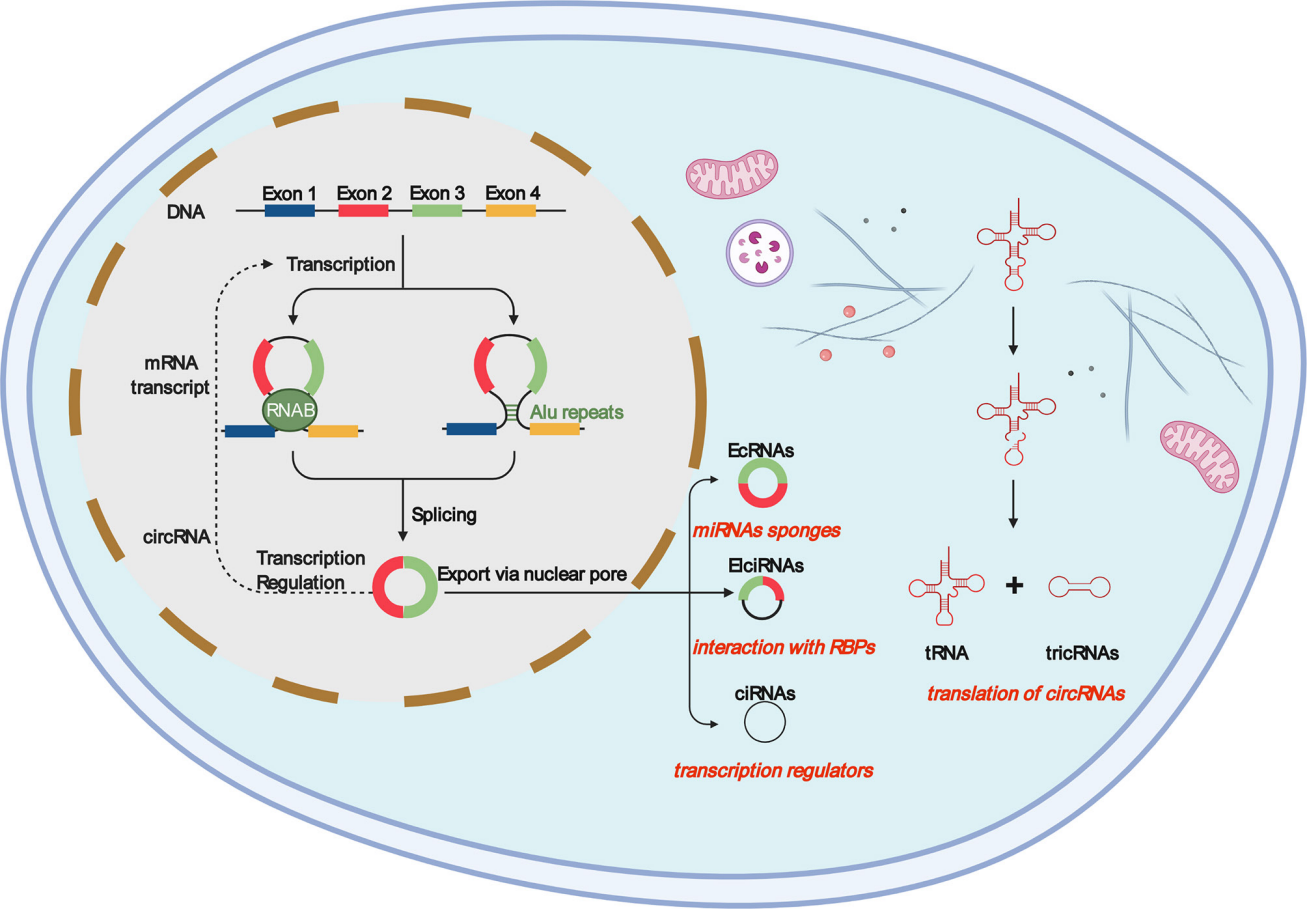
Most ecircRNAs are primarily produced from two or three exons of reverse splicing, in which the 3′ splicing donor of the pre-mRNA is covalent to the 5′ splicing receptor. Intron circular RNAs include circular intron RNAs (ciRNAs), excision of group I introns, excision of group II introns, excision of lariat introns, and excision of tRNA introns. Exon–intron circular RNA is a circular RNA in which exons and introns exist simultaneously. Internal repetitive sequences may play an important role in its generation, possibly similar to ecircRNAs. Intergenomic circRNAs contain two intron circRNAs, flanked by GT-AC splicing signals, which act as splicing donors and acceptors of circRNAs and form a complete circRNA. The resulting intron ends are joined by RtcB ligase to form a stable circRNA, named tricRNA.

circRNAs, with a closed-loop structure and high stability, are gene expression regulators that play a variety of regulatory roles in transcription, splicing, and chromatin interactions (52). The differences in the formation process and shape of the four circRNAs as well as the characteristics and functions of circRNAs are shown in **Figure 1** (53). circRNAs exhibit inherent conserved and environmental resistance stability due to their circular structure, presence in blood and peripheral tissues, and coexistence with exosomes and may be considered as potential biomarkers or therapeutic targets for a number of immune diseases (54). Most circRNA translation products have an impact on cancer progression or inhibition (49, 55), which leads to abnormal expression in various types of cancer (28), including colorectal cancer (56), hepatocellular carcinoma (5, 57, 58), gastric cancer (59, 60), acute promyelocytic leukemia (61), and breast cancer (62, 63). Listed in **Table 1** is the relationship between some circRNAs and tumors, suggesting that circRNAs are mainly related to inflammatory responses, including the interaction between cytokines and chemokines, and are a potential disease marker that can be used as promising biomarkers for diagnosis, providing a new therapeutic target for tumor treatment (65, 66).

**Table 1:** Some circRNA types in cancers.

Types	Expression	Cancers	miRNA sponges	Roles	Influence	References
circMTO1	Under-expressed	Hepatocellular carcinoma	miR-9	Tumor suppressor	It affects the expression of downstream P21 protein	(57)
circRNA cSMARCA5	Downregulated	Hepatocellular carcinoma	miR-181b-5p miR-17-3p	Tumor suppressor	It inhibits the proliferation and metastasis of the cancer by regulating TIMP3 expression	(5, 58)
circRNA circ-Ccnb1	Downregulated	Breast cancer	/	Cell death agent	It results in the induction of cell death in cancer	(62)
circGFRA1	Upregulated	Breast cancer	miR-34a ceRNA	Tumor suppressor	It regulates GFRA1 expression	(63)
circRNA f-circM9	Upregulated	Acute promyelocytic leukemia	/	Proto-oncogene	It contributes to cellular transformation in cancer	(61)
circRNA circ0006916	Downregulated	Lung cancer	miR-522-3p	Tumor suppressor	It inhibits cell proliferation by slowing down the cell cycle process	(64)
CiRS-7	Upregulated	Gastric cancer	miR-7	Tumor suppressor	It antagonizes the miR-7-mediated PTEN/PI3K/AKT pathway in gastric cancer	(59)
circ-ITCH	Upregulated	Gastric cancer	MiR-199a-5p	Tumor suppressor	It affects the EMT process of gastric cancer	(60)
circCCDC66	Upregulated	Colorectal cancer	miR-33b miR-93 miR-185	Proto-oncogene	It is associated with poor cancer prognosis	(56)

### TAMs

A tumor has a highly heterogeneous structure. Tumor cells interact with a variety of cells and factors, including immune cells and immune factors, to form a complex tumor immune microenvironment (67). The TME is a complex environment where tumor cells coexist with immune cells and other cells, blood vessels, signaling molecules, and the ECM and is the place where the immune system interacts with tumor cells (68). Exosomes are a component of the TME (69); they act as effective signaling molecules between cancer cells and surrounding cells that make up the TME (9, 70). Studies have found that circRNA molecules can be transferred to exosomes and are more abundant in exosomes than in cells, suggesting that they may be promising cancer biomarkers (9). Meanwhile, the TME can actively reprogram macrophage metabolism through the direct exchange of metabolites, cytokines, and other signaling mediators in cancer (71).

Macrophages are composed of many cell types with complex and delicate regulatory networks. The type, density, and location of macrophages, as well as other inflammatory infiltrates, have good prognostic value in various cancer types (72–74). Macrophages are key mediators of tissue homeostasis, while tumors upset this balance; macrophages can even become drivers of metastasis (72, 75). Macrophages are specialized phagocytes that differentiate from circulating classical monocytes after extravasation into tissues (76, 77) and express both activating and inhibiting receptors in the phagocytosis of opsonic or apoptotic cells (78). Macrophages can engulf a large number of pathogens and kill bacteria in cells (79–81). In addition to directly killing tumor cells, macrophages also serve as specialized antigen-presenting cells, which can present tumor cell-derived antigens on major histocompatibility complex (MHC) class I (82) and class II (83) molecules, thereby activating endogenous antitumor T cell responses, amplifying the therapeutic effect and reducing the risk of tumor cell escape due to antigen loss (84–86). TAMs are derived from bone marrow-derived monocytes and tissue macrophages that are recruited into and fill the TME, promoting the spread and diffusion of cancer cells (87–90). TAMs are key cells that generate immunosuppressive tumor microenvironments by producing cytokines, chemokines, and growth factors and triggering T cells to release inhibitory immune checkpoint proteins. TAMs can directly help tumor cells migrate through the paracrine ring between macrophages and tumor cells, which involves macrophages secreting epithelial growth factor (EGF) family ligands and tumor cells secreting CSF1, to improve the invasive characteristics of tumor cells (91).

**Figure 2:**
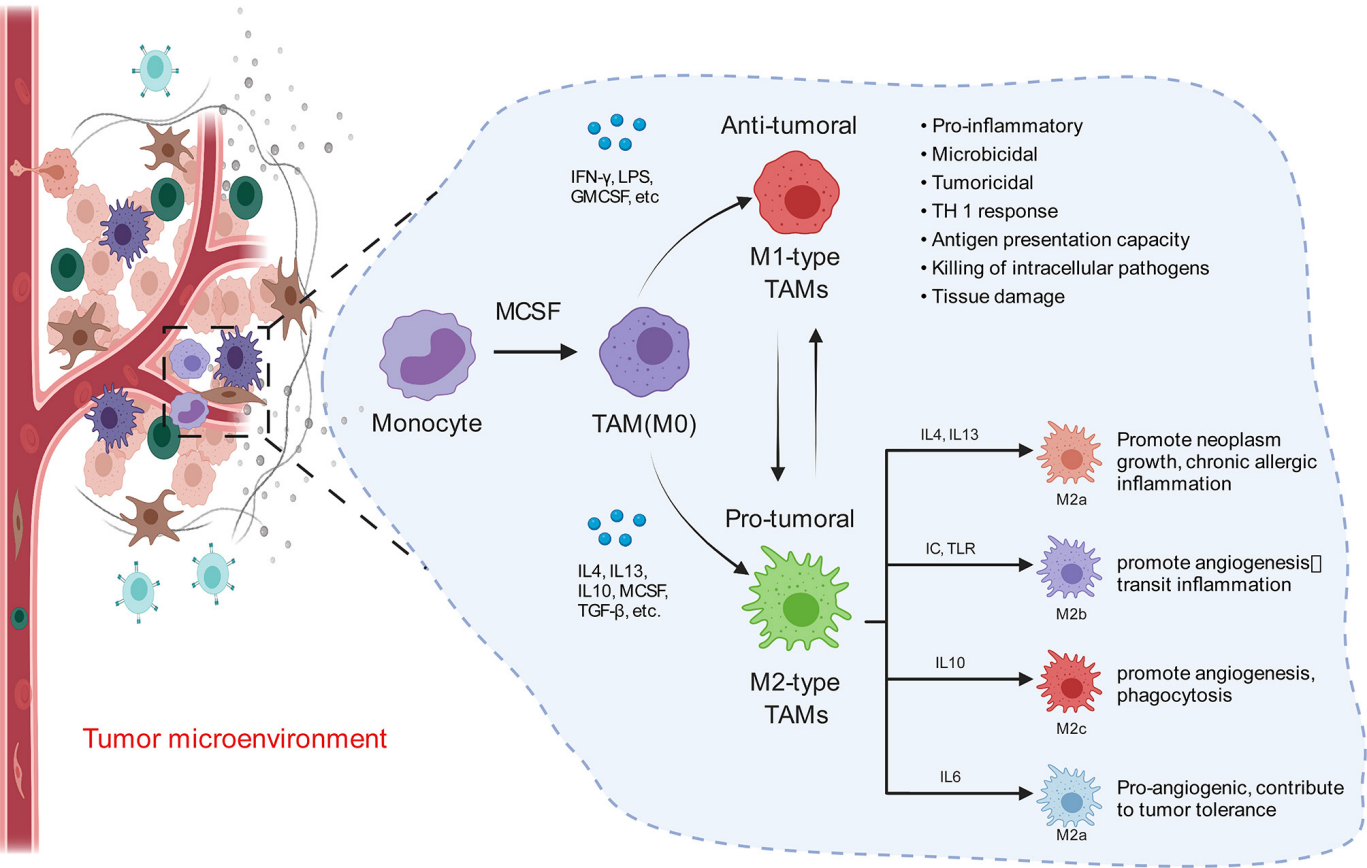
Macrophages in the tumor microenvironment become tumor-associated macrophages (M0) once exposed to tumor substances (MCSF), and M0 can differentiate into M1 and M2. When activated by interferon -γ and lipopolysaccharides, M1 macrophages show bactericidal activity and express high levels of CD86, inducible nitric oxide synthase, and pro-inflammatory cytokines, such as interleukin (IL-6), IL-12, IL-23, and tumor necrosis factor. M2 macrophages are activated by Th2 cytokines IL-4 and IL-13 as well as various parasite-related signals, which can be divided into four subtypes (M2a, M2b, M2c, and M2d). They are mainly involved in inhibiting type I inflammation and promoting tissue repair and healing responses.

Consistent with macrophages, TAMs are also highly plastic (92) and adapt to microenvironmental changes by regulating cell metabolism and reprogramming phenotypes (93, 94). TAMs enhance tumor progression by promoting genetic instability, angiogenesis, fibrosis, immunosuppression, lymphocyte rejection, invasion, and metastasis and promote the inflammatory environment by secreting cytokines, such as interleukin-17 (IL-17) and interleukin-23 (IL-23) (95, 96). Many studies have shown that TAM infiltration is closely related to tumor cell proliferation and can express a variety of cytokines that stimulate tumor cell proliferation and survival, including EGF, platelet-derived growth factor, transforming growth factor-β1, hepatocyte growth factor, and epidermal growth factor receptor (97, 98). **Figure 2** shows that, once monocytes from peripheral blood are recruited into the tumor, the tumor environment rapidly promotes their differentiation into TAMs (96). Initially, monocytes and macrophages are recruited to the site of tumorigenesis. Under the guidance of different microenvironmental signals, macrophages can differentiate into two functional phenotypes, namely, classical activated macrophages (M1) and alternately activated macrophages (M2). In contrast to the antitumor effects of M1, M2 has anti-inflammatory and tumorigenic properties. M2 TAMs are predominant in progressive tumors and are important regulatory cells in the TME response (99, 100). The major event in the tumor microenvironment is the polarization of macrophages into the tumor-suppressor M1 or tumor-promoting M2 macrophages. Although there is considerable evidence that TAMs are predominantly M2-like macrophages, the mechanisms by which TAMs polarize into M1 and M2 macrophages remain unclear (101). TAMs exhibit the patterns of M1 and M2 macrophages, but these cells are known to have transcriptional profiles different from those of M1 or M2 macrophages (102). However, it is certain that TAMs are related to the occurrence and development of various tumors, such as breast cancer, prostate cancer, glioma, lymphoma, bladder cancer, lung cancer, cervical cancer, and melanoma (103–105). TAMs, which are abundant in most types of malignancies, can promote tumor angiogenesis, allowing cancer cells to escape from the tumor into the circulation and inhibit anti-tumor immune mechanisms (106, 107). Some studies have shown that CSF1, IL-4, IL-13, and IL-10 can promote the polarization of M1-like TAMs to M2-like TAMs in the TME (102). Under specific conditions, the transformation of M2-like TAMs into M1-like TAMs may lead to tumor regression (108). By releasing pro-inflammatory molecules, such as TNF-α and IFN-γ, activating TLR, and reducing anti-inflammatory factors (such as ARG1, TGF-β, and IL10), M1-like TAMs can promote the inflammatory response and antitumor activity of the TME (109). TAMs can antagonize, enhance, or mediate the antitumor effects of cytotoxic agents, tumor irradiation, antiangiogenic/vascular injury agents, and checkpoint inhibitors (73, 110–113).

## The Relationship Between circRNAs and Macrophage Polarization

**Figure 3:**
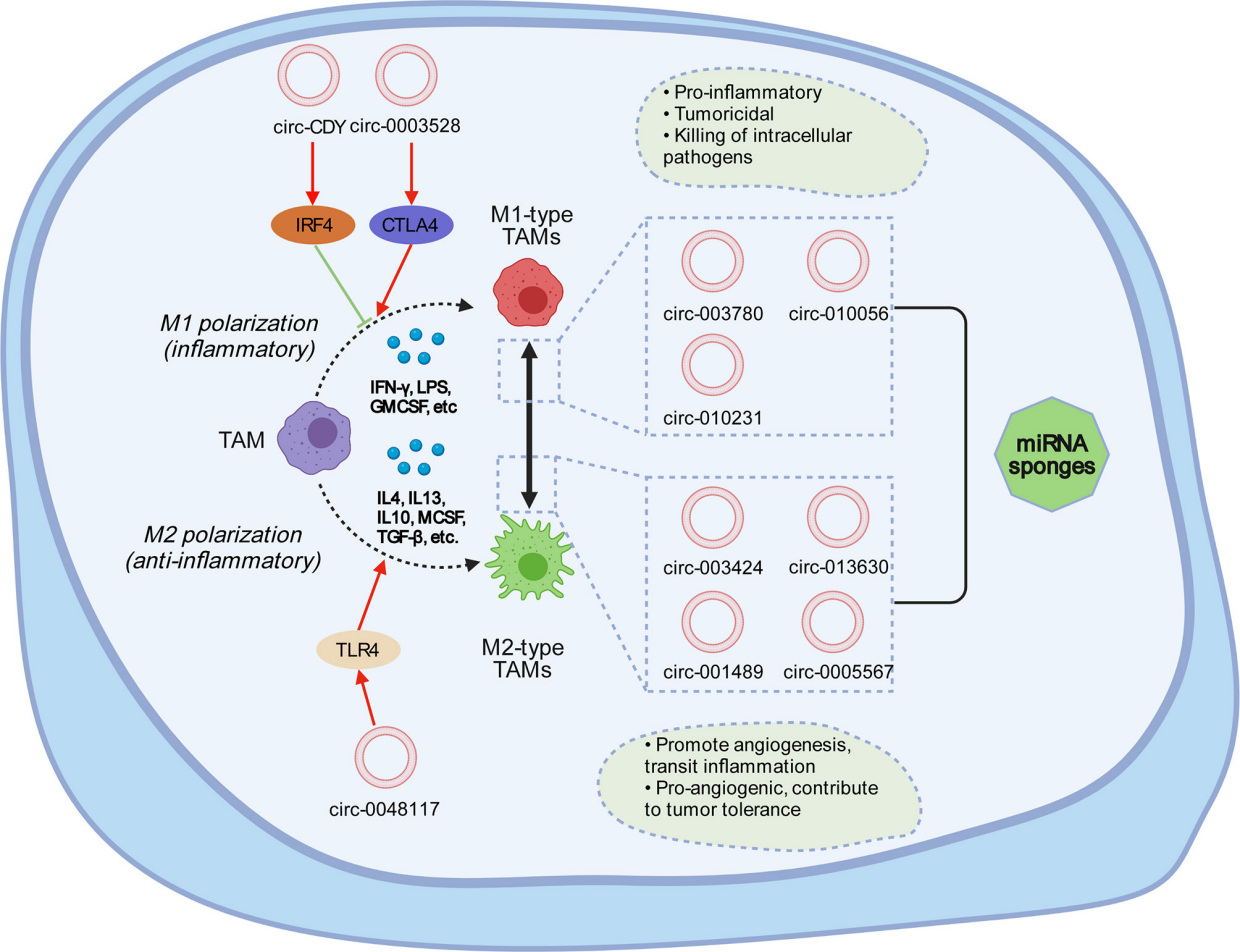
Tumor-associated macrophages can usually be divided into M1 type and M2 type. M1 macrophages are generally activated through interferon-γ and lipopolysaccharides, which mainly secrete pro-inflammatory factors and play a vital role in the early stage of inflammation. M2 macrophages are activated by Th2 cytokines, such as IL-4, IL-13, and the immune complex, express and inhibit the inflammatory factors, and play a role in inhibiting inflammatory response and tissue repair. circRNA can affect the mutual transformation between M1 and M2. circRNA-0003528 induces M1 macrophage polarization by activating CTLA4. circRNA-0048117 promotes the polarization of M1 to M2 macrophages by promoting the expression of TLR4. circ-Cdy can affect M1 polarization through IRF4. In M1 polarization, the expressions of circ-003780, circ-010056, and circ-010231 are upregulated, while the expressions of circ-003424, circ-013630, and circ-001489 are downregulated; the opposite is true for M2 polarization. circ-0005567 can inhibit M1 polarization and promote M2 polarization.

In primary tumors, TAMs have an M1-like phenotype and can eliminate certain immunogenic tumor cells (114). However, the TME can induce the M2-like polarization of TAMs, which is the cause of the formation of primary carcinoma (111, 115). Studies have demonstrated the relationship between circRNAs and macrophages—for example, circASAP1 can act as a competing endogenous RNA (ceRNA) of miR-326 and miR-532-5p to mediate TAM infiltration, and circRNA-CDR1as may be crucial for tumor tissue immunity and cell penetration, such as CD8^+^ T cells, activated natural killer (NK) cells, and M2 macrophages (116). circ-ASAP1 can mediate TAM osmosis by regulating the miR-326/miR-532-5P-CSF-1 pathway (117). There is no specific mechanism elucidated in this respect, which suggests that the next step is to study how circRNAs interact with tumor-associated macrophages. Studies have shown that circRNAs can regulate macrophage differentiation and polarization, while the pathways regulating macrophage polarization are not completely clear. Several molecules are involved in this process (**Figure 3**), such as interferon regulatory factor (IRF) and signal transducer and activator of transcription (STAT) (118, 119). Lipopolysaccharide stimulation (LPS) stimulates M1 macrophages by inducing STAT1-α and STAT1-β and interacting with the receptor TLR-4 (120). circPPM1F can play an active role in the LPS-induced activation of M1 macrophages through the circPPM1F–HUr–PPM1F–NF-κB axis (121), and circCdy can promote M1 polarization by inhibiting the entry of IRF4 into the nucleus (122). CSF-1, IL-4, IL-10, TGF-β, and IL-13 are conducive to the polarization of the M2 subgroup (123, 124). The overexpression of has-circ-0005567 inhibits the polarization of M1 macrophages and promotes the polarization of M2 macrophages (125). Compared with M2-type macrophages, circRNA-003780, circRNA-010056, and circRNA-010231 are upregulated in M1 macrophages. However, the expression of circRNA-003424, circRNA-013630, circRNA-001489, and circRNA-018127 is downregulated (126). Studies have shown that, in general, M1 is expressed in bones, the brain, and other adult tissues, and M2 is expressed in embryonic development stages, undifferentiated tissues, and tumors (127). circ-0048117 can regulate toll-like receptor 4 (TLR4) by acting as an miR-140 sponge to promote the polarization of M2 macrophages. Studies have confirmed that the activation of TLR4 on the surface of macrophages plays an important role in macrophage differentiation (128). TLR4 is strongly expressed in lung cancer TAMs and promotes the transformation of TAMs to M2-type macrophages by promoting the oxidative phosphatizing process in mitochondrial metabolism and inhibiting the glycolysis pathway (129). circRNA-0003528 promotes tuberculosis-associated macrophage polarization by upregulating the expression of cytotoxic T-lymphocyte-associated protein 4 (130).

Although most studies have found that circRNAs are more closely related to M2 polarization, the specific mechanism between circRNAs and M2 is not clear. Under the influence of tumor-derived factors, TAMs can secrete a series of cytokines, including IL-10, TGF-β, and prostaglandin 2 (PGE2), to further inhibit T cell-mediated immune responses and establish a self-proliferative immunosuppressive TME (72, 74). These cytokines can also affect circRNA expression. Accordingly, interleukin-10 (IL-10) can also inhibit the function of a variety of immune cells and the expression of anti-inflammatory macrophages; additionally, IL-10 can induce Tregs. Besides these, the abnormally expressed extracellular circRNA may induce Treg cells and interact directly with immune factors to mediate immune activity and facilitate intercellular communication (131, 132). IL-10 produced by TAMs can inhibit the function of antigen-presenting cells and then block the function of T cell effects, such as cytotoxicity, to inactivate the anti-tumor response, which leads to the downregulation of circRNAs related to the tumor response, thus promoting tumor growth (133). It was found that the transformation from M2-like TAMs to M1-like TAMs can lead to tumor regression under specific conditions (134). The mutual regulation between M1-like and M2-like TAMs is achieved by signal axes, such as STAT1/STAT6 and IRF5/IRF4, which are very important for the occurrence, development, and cessation of tumor inflammation (135). This may be a potential target for the regulation of M1-like and M2-like TAM transformation in clinical cancer immunotherapy. Promoting the conversion of M2 macrophages to the M1 phenotype can become a tumor treatment method (136).

Complex interactions exist between circRNAs and key counterparts in the TME (137), which can affect a variety of physiological and pathological activities, including tumor angiogenesis (138, 139). CAE is arranged on the inner surface of tumor blood vessels and lymphatic vessels, which can support angiogenesis and tumor neovascularization (140), while circ-CDR1as is positively correlated with the CAE infiltration level (18). Endothelial cells are the key to tumor angiogenesis, and the effect of circRNA on endothelial cells can affect tumor progression (141, 142)—for example, circ-IAR can destroy the tight junctions between endothelial cells, increase the permeability of vascular endothelial cells, and promote tumor metastasis (143). Some circRNAs can induce the expression of PD-L1 in the TME and mediate the regulation of tumor immunity—for example, the upregulation of PD-L1 mediated by has-circ-0020397 can lead to the inhibition of apoptosis and the acquisition of tumor immune escape in the TME (144). In short, tumor development is generally related to M2 polarization, and circRNA is also more closely related to M2 polarization and can act with related factors in the TME. The transformation from M2 to M1 can promote tumor regression, which suggests that we can further study key targeted circRNAs to inhibit M2 polarization and promote the transformation from M2 to M1 to treat tumors.

## circRNAs Are Associated With TAMs in Different Cancers

TAMs infiltrate most solid tumors in large numbers and contribute to tumor progression by stimulating proliferation, angiogenesis, and metastasis and providing anti-tumor immune barriers. Co-existing with circRNAs in the TME, TAMs may establish and reshape the extracellular matrix structure by allowing tumor cells to invade through the TME. circRNAs enable TAMs to interact with tumor cells or other stromal cells by secreting growth factors, cytokines, and chemokines (72, 76). Signals from tumor cells, lymphocytes, and stromal cells affect TAM function and diversity as well as the corresponding changes in circRNA expression (145, 146). circRNAs play a key role in the development and progression of human cancers such as lung, liver, breast, and colon cancers. Because of the large number of circRNAs, their functions may be complex and different from each other, so the functions and mechanisms of most circRNAs, such as has-circ-0014235, have not been fully identified, and little is known about the existence or effect of circRNA modification. The specific interactions between circRNAs and TAMs are also unknown. In TAMs, circPTK2 is mainly expressed in the tumor invasion frontier (M1-rich area) and stroma (M2-rich area). In contrast, circHIPK3 is mainly expressed in M2, located in the tumor nest and surrounding tumor invasion, suggesting that circRNA is closely related to tumor pathology and prognosis (147). Meanwhile, circRNAs have been found to be enriched in exosomes (4). circRNAs can specifically bind to tumor-specific miRNAs or mRNAs in exons and can be used as new tumor antigens to regulate the immune response (141). Some circRNAs can be detected in exosomes from the serum, urine, and tumors. Exosome circRNAs may be involved in cell growth, angiogenesis, epithelial–mesenchymal transformation, and targeted therapy (148, 149)—for example, circFBLIM1 carried by serum exosomes can be transferred to HCC cells, thereby promoting disease progression and suggesting that circRNA may be a biomarker of various diseases (150). In colorectal cancer, the exons secreted by M2 macrophages highly express miR-21-5p and miR-155-5p to regulate the migration and invasion of colorectal cancer cells (151). circ-BACH1 (has-circ-0061395) is significantly upregulated in HCC tissues, and p27 inhibition is regulated by HUR, which reduces circ-BACH1 to inhibit the proliferation and increase the apoptosis of HCC cells (152). Studies have shown that circASAP1 promotes the proliferation and invasion of HCC cells by regulating miR-326/miR-532-5p-MAPK1 signaling and regulates the infiltration of tumor-associated macrophages by regulating the miR-326/miR-532-5P-CSF-1 pathway (117).

High TAM infiltration is associated with a low overall survival rate in breast, gastric, oral, ovarian, bladder, and thyroid cancers but is not associated with low overall survival in colorectal cancer (CRC) (153). In bladder cancer, circPTK2 promotes the proliferation and migration of bladder tumor cells through the interaction of M2 tumor-associated macrophages (154), while circHIPK3 has been found in CRC by activating the downstream Bcl/Beclin 1 signaling pathway in the TME, thus promoting the growth and differentiation of TAMs (155). circRNA-002178 can act as a ceRNA and induce T cell depletion in lung adenocarcinoma by promoting PD-L1/PD1 expression in cancer cells with spongy miR-34 (156), which affects the polarization process of tumor-associated macrophages, as shown in **Figure 3**. Mutated p53 cancer cells can be re-transformed from macrophages to TAM through miR-1246 (157), which is beneficial for anti-inflammatory immunosuppression and the increased activities of TGF-β, while it also affects the expression of related circRNAs (158).

In TAMs, circ-0061395 competitively combines with miR-877-5p to improve the expression of PIK3R3 and promote the degree of malignancy of HCC cells. The inhibition of circWHSC1 *in vitro* can inhibit the proliferation and metastasis of HCC cells and inhibit tumorigenesis *in vivo* (159). Other studies have shown that circWHSC1, as a sponge for miR-142-3p, directly targets HOXA1, which inhibits the polarization of TAMs, while inhibiting miR-142-3p can improve the effects of circWHSC1 gene knockdown on the proliferation and metastasis of HCC cells. The overexpression of miR-142-3p inhibits the growth and motility of HCC cells, and the elevation of HOXA1 reverses this effect (160). The overexpression of circMCTP2 in gastric cancer can restore MTMR3 expression in gastric cancer cells to cisplatin through sponging miR-99a-5p, which also affects the process of macrophage reprogramming TAMs (161). Other studies have also shown that exosomal circRNA can promote cell growth and inhibit DNA damage (162). Experimental results have shown that circPPM1F can accelerate the activation of M1 macrophages and accelerate the apoptosis of islet cells in diabetic mice (121). Therefore, further studies of exosomal circRNAs will provide a new method for the diagnosis and targeted treatment of many diseases.

## Conclusion and Prospect on circRNAs and TAMs

With the development of next-generation sequencing technology, an increasing number of circRNAs have been discovered. circRNAs are a novel class of non-coding endogenous RNAs with closed-loop characteristics. At present, there is no evidence that circRNAs are directly related to the differentiation of tumor-associated macrophages, but some studies have shown that they can be transformed into endogenous RNA to participate in the differentiation process. However, not all circRNAs are positively correlated with tumor-associated macrophages, and the specific correlation between them deserves further study. In general, M1 macrophages promote inflammatory responses against invading pathogens and tumor cells, while M2 macrophages tend to exhibit an immunosuppressive phenotype that is conducive to tissue repair and tumor progression. The abundance of TAMs in tumors is often related to the acquisition of tumor-specific pathological features, such as immunosuppression, neovascularization, invasiveness, metastasis, and poor response to treatment, which indirectly suggests that TAMs may have a tumor-promoting function. TAMs may affect the expression of circRNAs in various cancers, such as lung cancer, liver cancer, colon cancer, and gastric cancer.

Tumor-associated macrophages, especially those that infiltrate tumor tissues, secrete various cytokines according to their polarity and play an important role in the occurrence, invasion, and metastasis of tumors. circRNAs, on the other hand, can specifically adsorb miRNAs to be used as competitive endogenous RNAs. By enhancing exon expression, circRNAs interact with the TME to establish an immunosuppressive environment and promote tumor cell proliferation, anti-apoptosis, invasion, and migration. Unfortunately, the cause and function of circRNA are not entirely clear; this is also true for the role of the specific mechanism between circRNAs and TAMs. However, previous studies have shown that there is little correlation between the two. Further research will help us delve into the role of circRNA and TAMs in tumor growth. The dynamic balance and interaction between TAMs and tumor cells play an important role in the occurrence and development of tumors. circRNAs can also affect TAM differentiation by influencing the TME, thus further affecting tumor growth and development. It was found that the expression of circRNAs in tumor tissues is not absolutely upregulated or downregulated; it may be upregulated in lung cancer but downregulated in breast cancer. Such contradictions make it difficult to connect the polarization of TAMs with the expression of circRNAs, but they show a correlation. Studies on the relationship between circRNAs and TAMs are still at a superficial stage, and there are not enough studies to prove a clear logical relationship between them. circRNAs have many small molecular subtypes, and the TAM polarization process involves many small molecular substances. Perhaps an algorithm can be developed to study the pairwise collocation or mixed collocation between the two to further reveal the relationship between them; this is a direction for future research. Further studies on the relationship between circRNAs and TAMs can help elucidate the role of circRNAs in the nervous system, cancer development, innate immune response, and other biological environments and diseases. circRNA is expected to become a new tumor marker and potential target, providing a new direction for tumor diagnosis and targeted therapy.

## Author Contributions

All authors listed have made a substantial, direct, and intellectual contribution to the work and approved it for publication.

## Funding

This work was partially supported by the National Natural Science Foundation of China (project no. 81602167), the Hunan Provincial Natural Science Foundation of China (project no. 2017JJ3494 and 2021JJ31100), and the Science and Technology Program Foundation of Changsha City (project no. kq2004085). This work was also supported in part by the National Natural Science Foundation of China (project no. 81803636 to XY) and Guangdong Basic and Applied Basic Research Foundation (project no. 2018A0303130329 to XY).

## Conflict of Interest

The authors declare that the research was conducted in the absence of any commercial or financial relationships that could be construed as a potential conflict of interest.

## Publisher’s Note

All claims expressed in this article are solely those of the authors and do not necessarily represent those of their affiliated organizations, or those of the publisher, the editors and the reviewers. Any product that may be evaluated in this article, or claim that may be made by its manufacturer, is not guaranteed or endorsed by the publisher.

## Abbreviations

APCs, antigen-presenting cells; CAE, cancer-associated endothelial cells; CAFs, cancer-associated fibroblasts; circ-CDR1as, cerebellar degeneration-related 1 antisense; circ-PAIP2, circRNA poly(A) binding protein-interacting protein 2; circRNA, circular RNA; circ-ZNF609CSCs, circRNA zinc-finger protein 609; CSC, cancer stem cells; CRC, colorectal cancer; CTLA4, cytotoxic T-lymphocyte-associated protein 4; ECM, extracellular matrix; EcRNA, exonic circRNa; EGF, epithelial growth factor; EGFR, epidermal growth factor receptor; ElciRNA, exon–intron circRNA; ceRNA, competing endogenous RNA; HCC, hepatocellular carcinoma; HGF, hepatocyte growth factor; IL-10, interleukin 10; IL-17, interleukin 17; IL-23, interleukin 23; IRF, interferon regulatory factor; LPS, lipopolysaccharide stimulation; MBLs, mannose-binding lectins; MHC, major histocompatibility complex; miR, microRNA; ncRNA, non-coding RNA; NGS, next-generation sequencing; PD-L1, programmed death ligand 1; PDGF, platelet-derived growth factor; PGE2, prostaglandin 2; Pol II, polymerase II; RBPs, retinol-binding proteins; STAT, signal transducer and activator of transcription; TAMs, tumor-associated macrophages; TGF-β1, transforming growth factor-β1; TLR4, regulate toll-like receptor 4; TME, tumor microenvironment.
